# Leadership in complex networks: the importance of network position and strategic action in a translational cancer research network

**DOI:** 10.1186/1748-5908-8-122

**Published:** 2013-10-11

**Authors:** Janet C Long, Frances C Cunningham, Janice Wiley, Peter Carswell, Jeffrey Braithwaite

**Affiliations:** 1 Centre for Clinical Governance Research, Australian Institute of Health Innovation, University of New South Wales, Level 1, AGSM Building, Kensington, Australia; 2 Centre for Primary Health Care Systems Research, Menzies School of Health Research, Charles Darwin University, Darwin, Australia; 3 School of Population Health, University of Auckland, Auckland, New Zealand

**Keywords:** Network analysis, Leadership, Health sector, Centrality, Brokerage, Key players, Research

## Abstract

**Background:**

Leadership behaviour in complex networks is under-researched, and little has been written concerning leadership of translational research networks (TRNs) that take discoveries made ‘at the bench’ and translate them into practices used ‘at the bedside.’ Understanding leaders’ opportunities and behaviours within TRNs working to solve this key problem in implementing evidence into clinical practice is therefore important. This study explored the network position of governing body members and perceptions of their role in a new TRN in Sydney, Australia. The paper asks three questions: Firstly, do the formal, mandated leaders of this TRN hold key positions of centrality or brokerage in the informal social network of collaborative ties? Secondly, if so, do they recognise the leadership opportunities that their network positions afford them? Thirdly, what activities associated with these key roles do they believe will maximise the TRN’s success?

**Methods:**

Semi-structured interviews of all 14 governing body members conducted in early 2012 explored perceptions of their roles and sought comments on a list of activities drawn from review of successful transdisciplinary collaboratives combined with central and brokerage roles. An on-line, whole network survey of all 68 TRN members sought to understand and map existing collaborative connections. Leaders’ positions in the network were assessed using UCInet, and graphs were generated in NetDraw.

**Results:**

Social network analysis identified that governing body members had high centrality and high brokerage potential in the informal network of work-related ties. Interviews showed perceived challenges including ‘silos’ and the mismatch between academic and clinical goals of research. Governing body members recognised their central positions, which would facilitate the leadership roles of leading, making decisions, and providing expert advice necessary for the co-ordination of effort and relevant input across domains. Brokerage potential was recognised in their clearly understood role of representing a specialty, campus or research group on the governing body to provide strategic linkages. Facilitation, mentoring and resolving conflicts within more localised project teams were spoken of as something ‘we do all the time anyway,’ as well as something they would do if called upon. These leadership roles are all linked with successful collaborative endeavours in other fields.

**Conclusions:**

This paper links the empirical findings of the social network analysis with the qualitative findings of the interviews to show that the leaders’ perceptions of their roles accord with both the potential inherent in their network positions as well as actual activities known to increase the success of transdisciplinary teams. Understanding this is key to successful TRNs.

## Background

### Introduction

If the wealth of biomedical research is to be translated into useful clinical practice and routine health decision-making, new knowledge must be taken out of the highly controlled laboratory environment and applied and understood in the messiness and complexity of actual patient and clinical service realities. Expertise and understanding from both arenas are essential to work out ways to make this happen. Translational research networks (TRNs) (Collaboration for Leadership in Applied Health Research and Care (CLAHRCs) in the UK [1] and health science alliances elsewhere) are a strategy to overcome the gaps between biomedical researchers and clinicians. They involve setting up an administrative structure to provide funding and shared resources, and a flatter, less hierarchical social structure than those found in individual hospitals or universitites so as to maximize collaboration, innovation and knowledge transfer across different disciplines, organisations, sites and specialties [2-9].

Despite the importance, little research assesses leadership behaviours in TRNs. Unlike university- or industry-based transdisciplinary teams, TRNs face problems of clinical service constraints and achieving outcomes, that while benefitting patients, may receive little academic kudos. Leaders of a network focused on translational research must manage differences in technical language, paradigms and approaches between researchers and clinicians. They need to find ways to promote strategic collaboration across disciplines, organisations and sites without overwhelming members with a deluge of new contacts [10].

### Leadership and relationships

One way of looking at leadership in these complex networks is to use a social network approach in which graph theory is used to quantify and explore the complex set of ties (relationships and interactions) between members [11,12]. Rather than considering the attributes or human capital held by members, such as expertise or skill sets, social network analysis considers members’ social capital. Here, we use social capital in the sense that Burt and Coleman use it; that is, to indicate the advantage that comes from a member’s individual positions within the overall configuration of their web of relationships [13,14]. Translational research networks are social networks that, in contrast to their formal structure, are defined by ties of communication or collaboration between members. Key players in social network terms are network members (‘actors’) who hold powerful or influential positions with high social capital [15], but it is not always true that key players are the mandated leaders [10,16]. Central actors are key players who interact with the most other actors, while brokers form links between isolated clusters, aiding cohesion through mediation behaviours [17]. Actors can hold both central and brokerage positions at the same time [12]. This study combined a social network survey with semi-structured interviews to explore the potential available to leaders through their network positions, and perceptions of their leadership roles. This research complements a larger research program exploring the role of key players in healthcare networks, and ultimately how their work can be supported and enhanced [18].

Centrality and brokerage have been suggested as positions that provide opportunities for effective leadership activities in complex networks [19-23]. Gray proposed a model of leadership for transdisciplinary teams, noting that leaders in central and brokerage positions within the network of members were best able to fulfil the various activities [19]. She grouped these activities into cognitive tasks, structural tasks and processual tasks. Cognitive tasks include communicating a vision of how members can overcome their discipline-specific assumptions and paradigms and learn to innovate and work together, as well as making decisions about such things as the composition of teams, which projects to support, and resource allocation. Structural activities involve co-ordination of the members and efficient information transfer, while processual activities are focused on ensuring that individual team interactions are constructive and any conflict is managed. Drawing on Stokol and colleagues’ review of geographically dispersed transdisciplinary teams [23], she also indicated that large and dispersed teams, such as the network studied here, require multiple leaders, a finding echoed by Greenfield and colleagues in a complex clinical setting [24]. These distributed leaders’ actions involved co-ordinating effort, ensuring information transfer across the network, and carrying out processual tasks for their local project groups.

### Barriers and enablers of translational research

Leaders’ behaviours and actions will be dependent not only on their position within the network but also on how they view the challenges facing the network. The literature reports four main challenges to the successful undertaking of translation research: structural, financial, intellectual and cultural factors. Structural challenges include the pressures of clinical service delivery, leaving clinicians little time for research [4,8,25-27]. Key financial challenges are the unreliability of funding [8,28], and lack of a recognised career path for translational researchers [4,28]. Intellectual challenges include the need to understand unique research designs [4,29], and legal and regulatory requirements [28,30]. Lack of standardisation of clinical trial protocols and data recording [2,3,27], and inability to access results from previous trials or research programs also hinders translation [2,3,8]. The formation of a network seeks to address these three factors by providing an interorganisational structure that can deliver reliable funding, institutional buy-in, research expertise, and supportive infrastructure.

The fourth challenge to translational research effectiveness is the mediating role of cultural factors. One manifestation is the differing paradigms, language and *modus operandi* of scientists and clinicians, which Dauphinée and Martin call the new ‘two cultures’ [8] after C.P. Snow’s use of the phrase to describe the lack of understanding between science and the arts [31]. These differences need to be managed to prevent miscommunications and team conflict as well as to smooth the dissemination of findings once teams have completed their projects [8,25,28,32]. Centrally located leaders who have relevant expertise and a vision of how successful translational research is achieved [23], or who can act as influential opinion leaders for the adoption of new findings, or who use their positions as brokers between the two cultures are well placed to mediate and smooth members’ interactions [19].

### Aim

Given the identified importance of leaders in TRNs and the limited empirical research of this area, the aim of this study was to identify how mandated leaders of a TRN use their network position to influence. This aim was advanced by collectively answering three questions: Firstly, do the formal, mandated leaders of this TRN hold key positions of centrality or brokerage in the informal social network of collaborative ties? Secondly, if so, do they recognise the leadership opportunities that their network positions afford them? Thirdly, what activities associated with these key roles of centrality and brokerage do they believe will maximise the TRN’s success and do these activities accord with Gray’s leadership model?

## Methods

### Setting

The setting is a translational cancer research network (hereafter the TRN) in Sydney, Australia, with 68 foundation members: clinicians based primarily in hospitals, and university-based biomedical and health services researchers. The TRN established a 14-member governing body to provide leadership and co-ordination of the network, including allocation of funds for 12-month projects and conference, travel and student grants. The governing body is made up of the network director, network manager, five senior leads in the disciplinary domains of basic science, health systems, clinical practice, primary health and pathology, and seven other members selected for their key knowledge or experience. Four members are primarily clinicians, six are researchers and three clinician-researchers with joint university and hospital appointments. The overarching goal of the network was ‘Taking science to practice’ and focussed on translating new, clinically proven knowledge of cancer processes, diagnostic or treatment techniques into routine clinical practice and health decision-making [6,27].

The TRN officially commenced in July 2011, and formal collaborations between members began in January 2012. Data collection for this study commenced in early 2012 at a time when there had been little official TRN contact between members. However, the TRN is embedded in a pre-existing and complex interorganisational network of long-standing research and teaching arrangements. TRN activities and funded projects do not displace existing and ongoing research such as National Health and Medical Research Council funded projects. At its inception, the TRN was already a collaborative effort with core group members who prepared and submitted the proposal to the funding body. Ethics approvals for the interview and online survey were obtained from the University of New South Wales (HREC 09085), appropriate regional health authority and site-specific committees, and all participants gave written consent.

### Social network data

All 68 members of the TRN were invited to complete an on-line survey in March 2012 [18]. Respondents were given a list of all members showing their name, job title and primary place of work and asked to indicate which members they knew before the TRN commenced. They were asked to select one of four categories to describe the type of relationship: collaborated with (*e.g*., on a funded project), worked with (*e.g*., shared care of patients or in the same research group), socialised with (*i.e*., outside of work), or knew by professional reputation (*e.g*., familiar with their published work, colleague of a colleague). As social ties were scant and were all reciprocated with either ‘collaborated’ or ‘worked with’ ties, all answers were combined to produce the Whole TRN Collaboration Network. Key player parameters were calculated for each member using UCInet v6 social network analysis software [33], and network diagrams were generated using NetDraw [34].

### Qualitative data

Semi-structured interviews were conducted with all 14 governing body members in early 2012. A total of 12 interviews were conducted face-to-face and two over the phone, lasting 30 to 45 minutes each. All interviews were recorded, transcribed, and qualitative data analysed with the assistance of NVivo 10 software.

The interview examined perceptions of the participant’s role and the activities he or she believed would achieve that role. A combination of deductive and inductive approaches was employed via a selection of open-ended and prompted questions. Opportunities were given to participants to talk freely about unanticipated challenges inherent in the new TRN structure. Interviews were conducted by a single researcher (JL) who had a background as a clinician, scientist, and health services researcher. Care was taken during interviews to ensure rigour through the use of structured, standardised questions and by checking participants’ meaning as needed (by asking reflexive questions) to ensure clarity of interpretation. Concepts and themes were coded independently by two reviewers (JL and JW) and finalised using discussion.

The first section of the interview asked participants to describe the network’s main objectives and the challenges they faced in achieving them. Other questions asked how they would measure network success formally and informally.

The second section asked open-ended and unprompted questions about the participant’s role within the network up until now and in the future. Participants were then shown a list of activities that have been associated with central and brokerage positions [35], and which Gray equates with successful leadership in complex networks (see Table 1). Participants were asked to indicate which ones they were doing now or expected to do in the future to further the aims of the network, and to comment on their choices.

**Table 1:** Central leadership positions, brokerage positions and the activities associated with each

**Network role**	**Position**	**Role**	**Activity**	**Comment**
**Central actor**	Interacts with the most other members	Leader	Leading, making decisions, co-ordinating, communicating	The actor interacts with the most other actors [15,36]
	High degree	Expert, opinion leader	Leading, giving expert advice, mentoring, communicating	The actor is a credible source of information and can lead change [37,38]
**Broker**	Links other who are not linked themselves	Boundary spanner, bridge, liaison	Representing, advocating, being a go-between, communicating	The actor links to a person or group outside of the network [17,39]
	High betweenness, high effective size	Bridge, broker	Linking, being a go-between, communicating	The actor facilitates collaboration between actors within the network [40,41]
		Knowledge broker, mediator, cultural boundary-spanner	Providing expert advice, resolving conflicts, facilitating, interpreting, communicating	The actor stands between other network members and facilitates the interaction [19,42]
		Gatekeeper	Controlling the flow of information or resources, communicating	May be positive (stopping unnecessary overload) or negative (impeding access; setting up inequalities) [38,39]

*Note.* Activities listed in the column marked ‘Activities’ were shown to the governing body members as part of the interview and asked: ‘Which of these activities are you doing now or expect to do in the future to further the aims of the network?’

## Results

### Social network analysis

**Figure 1:**
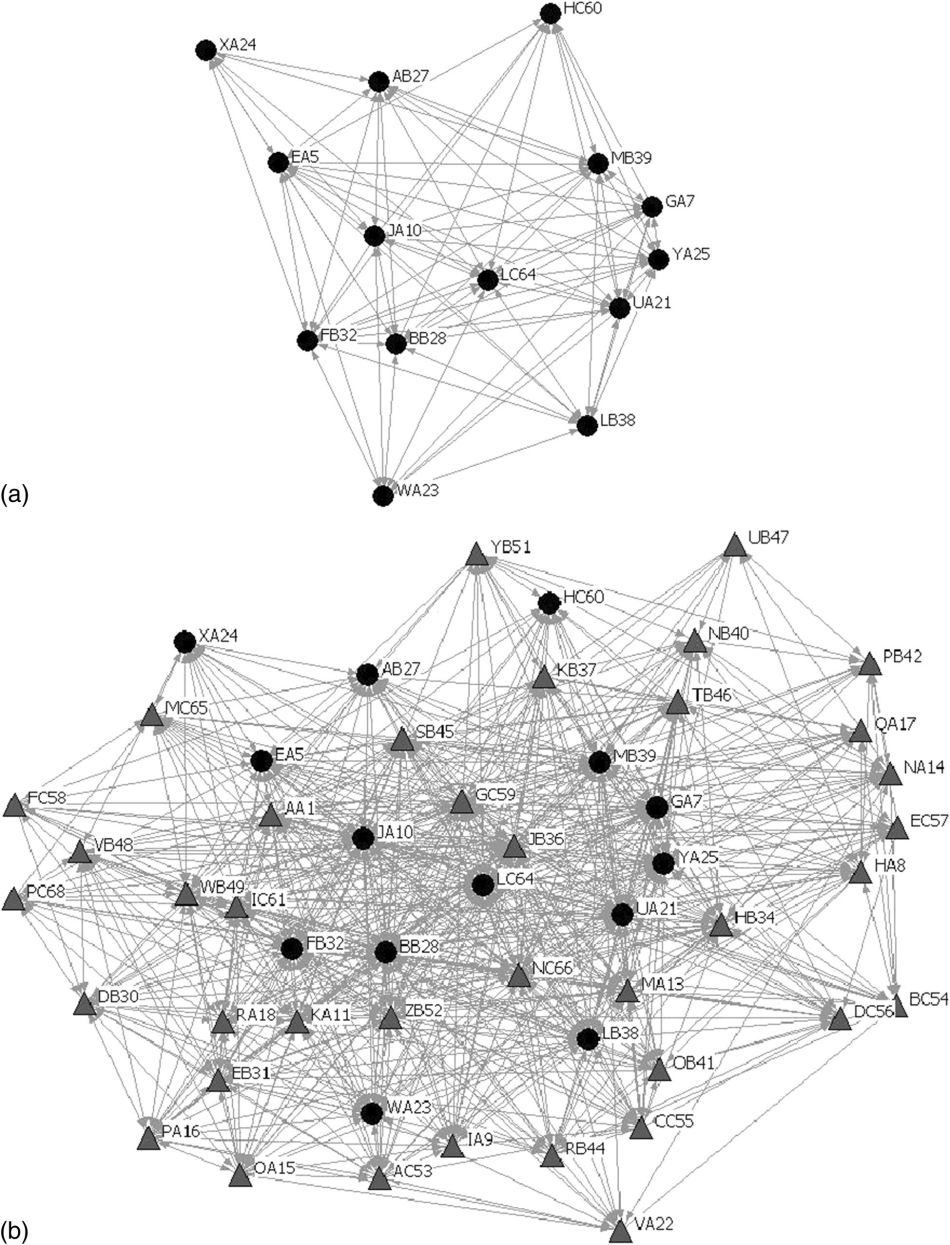
**(a). Social network diagram of governing body members answering the question: ‘Which other governing body members did you know before the TRN started?’ (b).** Social network diagram of whole TRN answering the question: ‘Which members did you know before the TRN started?’ Governing body members are black circles; other members are grey triangles.

The governing body was shown to be a well-connected group with positions conveying high centrality and brokerage potential in the Whole TRN Collaboration Network. Social network graphs are shown in Figure 1(a) and 1(b). The density of governing body members is 0.62, meaning that 62% of all possible linkages are present (Figure 1(a)). There were no isolates: members not already known to at least one other member.

**Figure 2:**
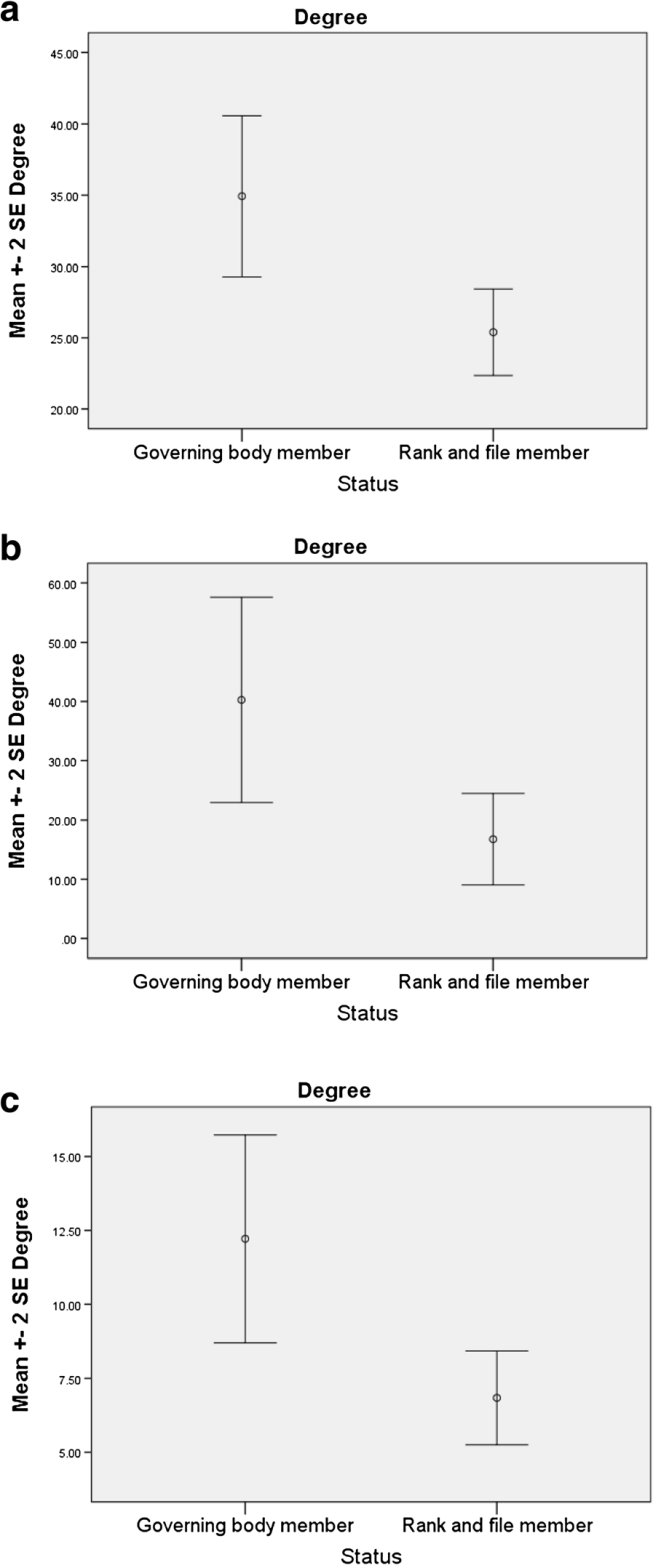
(a) to (c): Comparison of key player measures of governing body members and rank and file TRN members.

Independent sample t-tests were used to compare mean degree (a measure of centrality), mean betweenness, and mean effective size (measures of brokerage potential [17]) of govering body members and other network members. The t-tests were statististically significant across all three measures (see Table 2 and Figure 2(a) to (c)), showing that governing body members held more key positions and had a higher social capital than did their other colleagues. Moreover, eight of the nine members with the highest indegree, and eight of the eleven members with the highest betweenness centrality, were members of the governing body.

**Table 2:** Results of t-tests comparing mean degree, betweenness and effective size of governing body members and other members of the TRN

		**N**	**Mean**	**Standard deviation**	**t (df)**	**Significance**
Degree	Governing body	14	34.93	10.57	3.15	0.003
	Other members	38	25.39	9.35	(50)	
Betweenness	Governing body	14	40.27	32.36	2.85	0.006
	Other members	38	16.78	23.83	(50)	
Effective size	Governing body	14	12.22	6.57	3.19	0.002
	Other members	38	6.84	4.90	(50)	

### Interview data

All the participants described the main objective of the TRN as the translation of research into clinically useful outcomes, echoing the network’s vision statement. A total of 9 of the 14 participants spoke in terms of network outcomes that would directly affect the care that patients receive, while others spoke of engaging and co-ordinating people, providing research infrastructure, driving dissemination of findings, and facilitating collaborative links.

### ‘What are the challenges for the TRN?’

Ten participants mentioned the presence and persistence of silos - fragmented, insular and sometimes competitive subgroups - as a significant challenge. For example: ‘I have seen as much rivalry amongst people working in [the same field] from different sites, different universities, as I have collaboration among them’ (Interviewee XA24, researcher).

Another significant challenge mentioned by the majority of participants was the mismatch of researcher and clinician expectations, paradigms and methods. For example: ‘A lot of research is driven by academic parameters … winning grants and publishing papers… And the trouble is academic parameters have no relationship whatsoever to outcomes for patients. No-one cares’ (Interviewee WA23, researcher). A clinician stated, ‘[The TRN meeting] was scheduled at “professor time” which is a problem for people who have a clinical load’ (Interviewee UC60). Other issues raised were how to manage the diversity of stakeholders and how to engage those clinicians not already doing research.

### ‘What is your role?’

Two open-ended questions, ‘What has been your role so far in the TRN?’ and ‘What do you think will be your role in the future?’ were asked early in the interview. Ten of the 14 participants limited their answer to the former question to saying they had helped to draft (or had commented on) the initial grant proposal for the network and had attended the first governing body meeting. Four others had worked on TRN infrastructure projects such as setting up a database or recruiting translational research project officers. Future roles were far more diverse. People working on infrastructure projects saw that expanding or at least continuing. Four thought their role would be mainly providing expert advice, and one thought he could mentor and guide younger researchers’ projects in his field of expertise. Other answers included encouraging engagement among colleagues, advocating for their own research group, administering TRN funds, and leadership and governance of the network.

### Central actor roles and activities: leading, decision-making, co-ordinating, communicating, and opinion leading

All but two of the participants considered being on the governing body presupposed they would be undertaking leading and decision-making activities. The two that disagreed seemed to have different reasons for saying so. The first, a clinician-researcher who felt that the new way of accessing research grants from the funding body would disadvantage his research group, made a distinction between leading and making decisions: ‘Leading? Definitely not. I mean [the network director] is the leader but making decisions as opposed to leading, yes, because the charter of this executive is that they’re supposed to ultimately decide on the projects that get support. So, yes to that [making decisions] but no to that [leading]… The person who has the money controls the shots’ (Interviewee UA21, clinician-researcher). The other participant felt strongly that their role was more as an expert consultant. Thus, she stated, ‘I don’t think I’ll be a leader or making decisions really… My role is to provide input into the ideas that the researchers may have; whether it will work in the clinical setting’ (Interviewee GA7, clinician).

Both the formal written objectives and participants’ discourse on TRN objectives emphasised the importance of the central actor role of co-ordinating translational research effort across the sites and different disciplines. The work towards developing shared resources and support from project officers was discussed as a means of supporting that effort once collaborations had been started and was largely viewed as part of the role of the network manager and the project officers. Half of the participants agreed they would have a co-ordinating role, but it was generally qualified by limiting it to projects undertaken in their department or field of expertise, implying that they recognised their centrality in those areas but not network-wide. Seven participants said they would not, or would ‘probably’ not co-ordinate.

The network manager spoke at length about her role in co-ordinating members, the challenge it presented, and how she intended to achieve it: ‘I have made an effort to see almost all of the [governing body] members at least once at their location… I’ve had email contact with everyone on this list [68 network members]’ (Interviewee JA10, research manager).

Increasing the co-ordination and collaboration of siloed researchers and clinicians as a network objective was mentioned by 10 participants and was seen as a significant challenge and an important but difficult goal to achieve. Five people spoke of how the TRN hoped to change the culture of research, reducing the impact of silos and making it easier to form interorganisational collaborations and novel ways for clinicians and academics to interact. Two clinicians and two researchers were sceptical that the current network configuration would be adequate to do this, however.

Every participant acknowledged a role of providing expert advice, either to the governing body or to members, and this was seen as a valuable asset. Knowing the institutions and broader contexts in which the network was situated was also considered ‘expert knowledge.’ A clinician stated, ‘The other reason I’m there [on the governing body] is because I have been here [Hospital #1] for 20 years. I actually know the place reasonably well and all the wheeling and dealing that gets done around a hospital’ (Interviewee LB38).

Mentoring of project teams as a role for the governing body members was mentioned by three people as a subject that had been discussed in the first governing body meeting. Two people insisted it was not part of their role, neither having current supervisory roles with students. For the other 12 participants, mentoring was accepted as crucially important in the goal to make translational research sustainable.

Communication is an essential activity associated with both leadership and brokerage roles. In total, 11 members said they would be communicating as part of their role. Maintaining focus was identified by three participants as a challenge to the network. Four participants saw their leadership role on the governing body as keeping important issues for the network in focus, such as producing patient-centred outcomes and reflection on their evolving role. Two participants stated that they saw their role on the governing body as one of challenging the others, which may also be labelled as a leadership role akin to opinion-leading. Both used the phrase ‘I’m the only one who can say it.’ As said by a reasearcher, ‘You have got to deliver something [for patients] that is serious. My role in the Council is to say those kinds of things… I think probably I’m the only person in the room who can say it because I’m a basic scientist … therefore that gives me the right’ (Interviewee WA23). Another researcher said, ‘I’ve said things that no-one else in the room would say like “are we really using the best evidence possible to deliver care to the patients, because our research tells us that there’s a lot of people who are not,” and you know already none of the clinicians in the room would have said that but I was able to’ (Interviewee EA5).

### Brokerage roles and activities: representing, bridging, mediating, resolving conflict, and gate keeping

While no participant used the word ‘broker’ in the open-ended question on roles in the TRN, all participants selected one or more brokerage activities from the list provided. Most participants saw their well-connected, central positions within their own department or research area as an asset for the network as it allowed them to take on brokerage roles and use these links for the network.

The terms of reference for the governing body explicitly stated that members were chosen for their ability to represent certain disciplinary skills and the key campuses involved. All participants were quite clear about their formal representative role on the governing body, and some participants also claimed to represent nurses, surgeons, pathologists and their own research groups. Some felt they had several representative roles: ‘[I have] dual roles: representing education and [Hospital #3]’ (Interviewee MB39, clinician).

Representation is a brokerage role in that the actor links the group they represent to the network. This is an important role because it gives the network access to a diverse and rich source of ideas and viewpoints yet without making it unmanageable with large numbers of people [13,40]. One of the network’s strategies was to ensure representation on the governing body from health services researchers (specialists in evidence-creation, implementation and evaluation), primary health researchers (often omitted as not hospital-based), and health economists (specialists in value for money, project viability and sustainability). A researcher stated, ‘Having a [TRN] that has this group of expertise available to it would really put it ahead of the pack’ (Interviewee EB5).

All participants were clear that their role would include brokerage in terms of facilitating actual research projects undertaken in their area, though several used the qualifying phrase ‘if I was asked.’ Words chosen for this role were: being a go-between, liaising or bridging. A clinician stated, ‘You know I might say look, go and see so and so; he might be able to sort that out’ (Interviewee LB38, clinician).

‘Resolving conflict within teams’ was interpreted as the inevitable part of any research collaboration, more about personalities involved than as a result of a mismatch of clinicians’ and scientists’ expectations. As stated by a clinician, ‘Resolving conflicts within research teams, yep. We do a certain amount of that unfortunately. There’s always little interpersonal things niggling away’ (Interviewee YA25). Participants were split between thinking it was their role (five participants) to resolve these types of conflicts, and thinking it was not (seven). Four people thought it was the role of the governing body or network director but not them individually.

As well as providing expert knowledge, three participants also described situations where their role was more like that of an interpreter or translator. All three were in highly specialised roles. For example: ‘I think we do have, like all disciplines, a set of jargon and a set of words that are used and it will be definitely part of my role to interpret that and explain that … also … looking at a set of results or issues and saying well, from my perspective this is how I would look at it’ (Interviewee XA24, researcher).

Gatekeeping was discussed by two participants in terms of the unpalatable governing body role of allocating research grants where they may have to ‘say no’ to their peers. Two other participants described themselves as people who open or slip through gates to facilitate access to resources. One clinician saw gatekeeping as a positive and useful role: ‘This person wanted to meet everybody and I said … that’s not appropriate. You need to tell me exactly what you want and then I can give you who you need to meet with. So I understand that I am the gatekeeper’ (Interviewee GA7, clinician-manager).

Another brokerage role not listed but discussed by two members was that of boundary spanning; *i.e*., linking to people outside of the TRN in order to improve TRN outcomes. The network manager discussed meeting with managers of other newly formed TRNs supported by the same funding body but which had a different structure. One of the other researchers said he was looking forward to consulting with a research group from another of these TRNs, as he felt the previous siloed and competitive culture of conducting research was changing.

## Discussion

We set out to answer three questions to illuminate a core problem in implementation science. The first asked: do the formal, mandated leaders of a TRN hold key positions of centrality or brokerage in the informal social network of collaborative ties? We have shown this to be so in this case. This is a contribution to the literature on the role of leaders in networks. While other studies have shown that appointed leaders do not always turn out to be key players in their networks when formally evaluated with social network analysis [10,20], it may well be that the pattern is different in TRNs. These are networks that have a specific purpose of applying evidence in practice. Consequently, the leaders chosen sit in key formal positions that affect both the questions that are researched, and the decision to apply new ideas in practice. Given the increasing use of TRNs, along with the limited empiricial focus on their collaborative functioning, this is a key contribution.

In relation to the second and third questions, participants did recognise and were very clear on their positions as being centrally involved in the web of member interactions and knowing the skills and expertise of members so that strategic linkages could be facilitated. The brokerage role of representing a domain such as pathology, a professional group such as nursing or an individual site, was seen in terms of co-ordinating effort across diverse areas and leveraging expertise from a range of stakeholders [35]. Activities associated with Gray’s leadership model were all recognised and included.

The significant challenge of bridging between clinical and research group silos was identified by participants and accords with other research on gaps and clusters in healthcare, translational research, and the academic sector [8,43,44]. Brokers are able to bridge these groups, often by ‘having a foot in each camp.’ Here, clinician-researchers with an academic position and a clinical position have an insight into both domains and understand the specialised language and tacit knowledge of each. This can foster trust and respect and facilitate interactions [19,42]. Almost all of the governing body members had previous experience of translational research, which also facilitates understanding through knowledge of the particular norms, routines and expectations of translational teams.

The acceptance of the processual tasks of facilitating and resolving conflict among research teams also accords well with Gray’s model of distributed leadership, even if there was some reluctance to offer their services without invitation [19]. Once again, the dual aspects of human capital and social capital in the leadership role are apparent. Human capital is used to understand the issues involved and social capital to act as an effective facilitator, conflict resolver, or go-between to direct team members to the help or resources they need.

Participant UA21’s distinction between decision-making and leading was an unexpected one and stood out from the rest of the participants’ answers. This clinician-researcher equated the TRN director’s ‘power’ with ‘holding the purse strings,’ saying that the director and their close associates were therefore the ones leading or ‘calling the shots.’ UA21 further indicated that changes in the administration of research funding through the TRN was likely to have a negative impact on their research group and was an untested and difficult model.

The TRN manager directed a great deal of effort on commencement in the position into communicating with every member, often visiting them in their own workplace to establish a rapport and better understand their needs. This recognition and investment in social capital meant that the manager was in a good position to leverage development of TRN resources and to facilitate members’ access to expertise and resources.

### Strengths, limitations and significance

This study was a full census, not a sample, so although the number of participants of the interviews was only 14, all appointed leaders of the TRN provided the full range of views. Further, while this study was based on a single TRN with a particular structure, the case study approach allows for a more detailed understanding of the interrelationships and perceptions of the governing board members. Little empirical research has been conducted on either the leadership roles in collaborative healthcare networks or brokerage roles in that context, and so this study provides insights into how leaders of other translational research or transdisciplinary endeavours may frame their roles.

## Conclusions

The governing body members were confirmed as key players by virtue of their position within the whole TRN network of members, showing their potential as both central actors and brokers. They were more centrally placed and interacted with more members on average than did the other members. They also acted as a bridge between members who did not know each other more often than most other members.

Participants had a clear understanding of the TRN’s objectives and vision, and this was well aligned with the written objectives. The open-ended questions on their perceived role elicited a range of answers, but apart from ‘encouraging engagement among my colleagues’ and boundary spanning, all the activities had been anticipated and were included on the prompt list. Overall, our expectations of the 14 mandated key players’ perceived roles were largely confirmed. Leading, making decisions, and providing expert advice were almost unanimously selected as part of the leadership role of a governing body member. Brokerage activities were also seen as a component of their role, although some brokerage activities were chosen more often than others. Representing a specialty, campus or research group on the governing body was nominated by everyone, with facilitation and mentoring research teams chosen by all but two. Although the term was never used, there was a strong perception they were acting as a ‘brain trust’ for the members in the sense that they were all experts and willing to give advice.

There seemed to be a distinction between roles that participants took on as part of the TRN that were just for the TRN – for example, making decisions on funding allocation, infrastructure and future directions – and facilitative roles that ‘we do all the time,’ like mentoring and troubleshooting problems within project teams. The TRN is a hybrid of a hierarchical, and a flatter, enclave style of network [45]. Stokol and colleagues report that the presence of multiple leaders and champions and a non-hierarchical membership that allows autonomy while encouraging participatory goal setting are all key factors for effective transdisciplinary teams [23]. Gray also notes this point and adds the importance of co-ordination between these multiple leaders or local champions [19]. Governing body members’ willingness to mentor and facilitate research teams (even if only ‘when required’) makes them local leaders and champions, as does the activity of encouraging engagement of colleagues not already engaged in translational research. At the same time, the governing body structure allowed co-ordination and the representation of discipline, specialty, campus or research group and was seen as a means of bridging the ‘two cultures’ (*i.e*., university-based researchers versus hospital-based clinicians) and making sure all views and expertise were considered [8]. Leaders’ perceptions of their role accord with factors known to increase the success of transdisciplinary or interorganisational teams: communicating the TRN’s vision, connecting and co-ordinating effort across diverse groups, facilitating local project teams, and ensuring there is understanding between the ‘two cultures.’

## Competing interests

The authors declare that they have no competing interest.

## Authors’ contributions

Protocol was developed by JL, FC, PC and JB. JL wrote the paper. All authors critically reviewed all drafts, and read and approved the final manuscript.

## References

[B1] Rowley E Morriss R Currie G Schneider J Research into practice: Collaboration for Leadership in Applied Health Research and Care (CLAHRC) for Nottinghamshire, Derbyshire, Lincolnshire (NDL) Implement Sci 2012 7 40 10.1186/1748-5908-7-40 22553966 PMC3441357

[B2] Field A Baxter K Terry SF From bench to practice to population health impact: barriers to realizing the public health and clinical promise of basic scientific discovery Genet Test Mol Biomarkers 2011 15 191 192 10.1089/gtmb.2011.1518 21428744

[B3] Mathew JP Taylor BS Bader GD Pyarajan S Antoniotti M Chinnaiyan AM Sander C Burakoff SJ Mishra B From bytes to bedside: data integration and computational biology for translational cancer research PLoS Comput Biol 2007 3 e12 10.1371/journal.pcbi.0030012 17319736 PMC1808026

[B4] Tageja N Bridging the translation gap - new hopes, new challenges Fundam Clin Pharmacol 2011 25 163 171 10.1111/j.1472-8206.2010.00903.x 21155875

[B5] Tenenbaum JD Whetzel PL Anderson K Borromeo CD Dinov ID Gabriel D Kirschner B Mirel B Morris T Noy N The Biomedical Resource Ontology (BRO) to enable resource discovery in clinical and translational research J Biomed Inf 2011 44 137 145 10.1016/j.jbi.2010.10.003 PMC3050430 20955817

[B6] Woolf SH The meaning of translational research and why it matters JAMA 2008 299 211 213 10.1001/jama.2007.26 18182604

[B7] Zerhouni EA Translational and clinical science: time for a new vision N Engl J Med 2005 353 1621 1623 10.1056/NEJMsb053723 16221788

[B8] Dauphinée D Martin JB Breaking down the walls: thoughts on the scholarship of integration Acad Med 2000 75 881 886 10.1097/00001888-200009000-00008 10995608

[B9] Goldblatt EM Lee W-H From bench to bedside: the growing use of translational research in cancer medicine Am J Transl Res 2010 2 1 18 20182579 PMC2826819

[B10] Cross R Parker A The hidden power of social networks: understanding how work really gets done in organizations 2004 Boston, Massachusetts: Harvard Business School Press

[B11] Wasserman S Faust K Social network analysis 1994 Cambridge: Cambridge University Press

[B12] Scott J Social network analysis: a handbook 2000 2 London: Sage

[B13] Burt RS Brokerage and closure: an introduction to social capital 2005 New York: Oxford University Press

[B14] Coleman JS Social capital in the creation of human capital Am J Sociol 1988 94 S95 S120 10.1086/228943

[B15] Borgatti S Identifying sets of key players in a social network Comput Math Organiz Theor 2006 12 21 10.1007/s10588-006-7084-x

[B16] Krackhardt D Hanson JR Informal networks: The company behind the charts Harvard Bus Rev 1993 71 104 111 10127036

[B17] Burt RS Structural holes: the social structure of competition 1992 Cambridge, Massachusetts: Harvard University Press

[B18] Long JC Cunningham FC Braithwaite J Network structure and the role of key players in a translational cancer research network: a study protocol BMJ Open 2012 2 doi: 10.1136/bmjopen-2012-001434 10.1136/bmjopen-2012-001434 PMC3383981 22734122

[B19] Gray B Enhancing transdisciplinary research through collaborative leadership Am J Prev Med 2008 35 S124 S132 10.1016/j.amepre.2008.03.037 18619392 PMC2542584

[B20] Cross R Borgatti S Parker A Making invisible work visible: using social network analysis to support strategic collaboration Calif Manage Rev 2002 44 25 46 10.2307/41166121

[B21] Chauvet V Chollett B Soda G Huault I The contribution of network research to managerial culture and practice Eur Manage J 2011 29 321 334 10.1016/j.emj.2011.06.005

[B22] Balkundi P Kilduff M The ties that lead: a social network approach to leadership Leadersh Q 2005 16 941 962 10.1016/j.leaqua.2005.09.004

[B23] Stokols D Misra S Moser RP Hall KL Taylor BK The ecology of team science: understanding contextual influences on transdisciplinary collaboration Am J Prev Med 2008 35 S96 S115 10.1016/j.amepre.2008.05.003 18619410

[B24] Greenfield D Braithwaite J Pawsey M Johnson B Robinson M Distributed leadership to mobilise capacity for accreditation research J Health Organ Manag 2009 23 255 267 10.1108/14777260910960975 19711782

[B25] Bristow R Recommendations for the future of translational radiobiology research: a Canadian perspective Radiother Oncol 2004 70 159 164 10.1016/j.radonc.2004.02.002 15028402

[B26] Mankoff S Brander C Ferrone S Marincola F Lost in translation: obstacles to translational medicine J Transl Med 2004 2 14 10.1186/1479-5876-2-14 15149545 PMC420261

[B27] Sung NS Crowley WF Genel M Salber P Sandy L Sherwood LM Johnson SB Catanese V Tilson H Getz K Central challenges facing the national clinical research enterprise JAMA 2003 289 1278 1287 10.1001/jama.289.10.1278 12633190

[B28] Carpenter S Carving a career in translational research Science 2007 317 966 967 10.1126/science.317.5840.966 17702947

[B29] Westfall JM Mold J Fagnan L Practice-based research - “blue highways” on the NIH Roadmap JAMA 2007 297 403 406 10.1001/jama.297.4.403 17244837

[B30] Marantz PR Strelnick AH Currie B Bhalla R Blank AE Meissner P Selwyn PA Walker EA Hsu DT Shamoon H Developing a multidisciplinary model of comparative effectiveness research within a clinical and translational science award Acad Med 2011 86 712 717 10.1097/ACM.0b013e318217ea82 21512360 PMC3102772

[B31] Snow C The Two Cultures and the Scientific Revolution 1959 Cambridge, England: Cambridge University Press

[B32] Ioannidis JP Materializing research promises: opportunities, priorities and conflicts in translational medicine J Transl Med 2004 2 5 10 10.1186/1479-5876-2-5 14754464 PMC343300

[B33] Borgatti S Everett MG Freeman LC UCInet for Windows: software for social network analysis 2002 6 Harvard: Analytic Technologies

[B34] Borgatti S NetDraw: graph visualization software 2002 Analytic Technologies: Harvard

[B35] Long J Cunningham FC Braithwaite J Bridges, brokers and boundary spanners in social professional networks: a systematic review BMC Health Serv Res 2013 13 158 10.1186/1472-6963-13-158 23631517 PMC3648408

[B36] Watts DJ Strogatz SH Collective dynamics of ‘small-world’ networks Nature 1998 393 440 442 10.1038/30918 9623998

[B37] Valente TW Pumpuang P Identifying opinion leaders to promote behavior change Health Educ Behav 2007 34 881 896 17602096 10.1177/1090198106297855

[B38] Valente TW Network interventions Science 2012 337 49 53 10.1126/science.1217330 22767921

[B39] Gould RV Fernandez RM Structures of mediation: a formal approach to brokerage in transaction networks Sociol Methodol 1989 19 89 126

[B40] Burt RS Structural holes and good ideas Am J Sociol 2004 110 349 399 10.1086/421787

[B41] Valente TW Fujimoto K Bridging: locating critical connectors in a network Soc Networks 2010 23 212 220 20582157 10.1016/j.socnet.2010.03.003 PMC2889704

[B42] Di Marco MK Taylor JE Alin P Emergence and role of cultural boundary spanners in global engineering project networks J Manag Eng 2010 26 123 132 10.1061/(ASCE)ME.1943-5479.0000019

[B43] McInnes E Middleton S Gardner G Haines M Haertsch M Paul CL Castaldi P A qualitative study of stakeholder views of the conditions for and outcomes of successful clinical networks BMC Health Serv Res 2012 12 49 doi: 10.1186/1472-6963-12-49 10.1186/1472-6963-12-49 PMC3325167 22373078

[B44] Braithwaite J Between group behaviour in health care: gaps, edges, boundaries, disconnections, weak ties, spaces and holes. A systematic review BMC Health Serv Res 2010 10 330 10.1186/1472-6963-10-330 21134295 PMC3004899

[B45] Goodwin N Perri Peck P Freeman T Posaner R Managing across diverse networks: lessons from other sectors. Report to the national coordinating centre for the NHS service delivery and organisation R&D programme 2004 London: London School of Hygiene and Tropical Medicine

